# Efficacy of Green Synthesized Nanoparticles in Photodynamic Therapy: A Therapeutic Approach

**DOI:** 10.3390/ijms241310931

**Published:** 2023-06-30

**Authors:** Mehak Zahra, Alexander Chota, Heidi Abrahamse, Blassan P. George

**Affiliations:** Laser Research Centre, Faculty of Health Sciences, University of Johannesburg, P.O. Box 1711, Doornfontein 2028, South Africa; mehakzahra.zm@gmail.com (M.Z.); chotatimzy@gmail.com (A.C.); habrahamse@uj.ac.za (H.A.)

**Keywords:** cancer, photodynamic therapy, nanotechnology, nanoparticles, cell death

## Abstract

Cancer is a complex and diverse disease characterized by the uncontrolled growth of abnormal cells in the body. It poses a significant global public health challenge and remains a leading cause of death. The rise in cancer cases and deaths is a significant worry, emphasizing the immediate need for increased awareness, prevention, and treatment measures. Photodynamic therapy (PDT) has emerged as a potential treatment for various types of cancer, including skin, lung, bladder, and oesophageal cancer. A key advantage of PDT is its ability to selectively target cancer cells while sparing normal cells. This is achieved by preferentially accumulating photosensitizing agents (PS) in cancer cells and precisely directing light activation to the tumour site. Consequently, PDT reduces the risk of harming surrounding healthy cells, which is a common drawback of conventional therapies such as chemotherapy and radiation therapy. The use of medicinal plants for therapeutic purposes has a long history dating back thousands of years and continues to be an integral part of healthcare in many cultures worldwide. Plant extracts and phytochemicals have demonstrated the ability to enhance the effectiveness of PDT by increasing the production of reactive oxygen species (ROS) and promoting apoptosis (cell death) in cancer cells. This natural approach capitalizes on the eco-friendly nature of plant-based photoactive compounds, offering valuable insights for future research. Nanotechnology has also played a pivotal role in medical advancements, particularly in the development of targeted drug delivery systems. Therefore, this review explores the potential of utilizing photosensitizing phytochemicals derived from medicinal plants as a viable source for PDT in the treatment of cancer. The integration of green photodynamic therapy with plant-based compounds holds promise for novel treatment alternatives for various chronic illnesses. By harnessing the scientific potential of plant-based compounds for PDT, we can pave the way for innovative and sustainable treatment strategies.

## 1. Introduction

Cancer is a disease with a complex and diverse nature that originates from the uncontrollable growth of atypical cells within the body [1]. It is regarded as a major public health problem worldwide and a leading cause of death [2]. The development of cancer is a multistep process that involves genetic alterations, epigenetic changes and interactions with the tumour microenvironment [3]. It is estimated that 1,958,310 new cancer cases and 609,820 cancer deaths will occur in the United States in 2023 [4]. According to GLOBOCAN, the international agency for research on cancer, the worldwide occurrence of cancer is estimated to total 19.3 million cases in 2020 and is predicted to increase to 30.2 million cases by 2040 [5]. Several risk factors can be attributed to the increase in the occurrence and fatality rates of cancer. Various factors can contribute to certain health conditions, such as alcohol consumption, advanced age, hormonal imbalances, genetic susceptibility, and poor lifestyle choices [6]. The predominant treatment approaches employed in the management of breast cancer comprise surgical procedures, radiotherapy, chemotherapy, and hormonal therapy [7]. Typically, patients with cancer may undergo surgery and radiotherapy as the initial or standard treatment, followed by adjuvant therapy which may consist of either hormonal therapy or chemotherapy [8]. Conventional cancer treatment modalities, such as chemotherapy, radiation therapy, and surgery, have certain limitations that can impact patient life. Chemotherapy is known to cause significant side effects such as fatigue, hair loss, and nausea. Unfortunately, it can also destroy healthy cells while not effectively targeting cancer cells [9]. Chemotherapy regimens that are frequently utilized can carry some risks, both in the short and long term, even if they are relatively minor. This is particularly relevant for breast cancer patients, who tend to be older and may have other health issues. In addition, older women may face a greater potential for complications that result in decreased functionality as a result of receiving adjuvant chemotherapy [10].

When considering radiation therapy, it is important to note that it can lead to several adverse effects. These may include skin irritation, fatigue, and harm to healthy tissue, while the therapy may not be effective for certain types of cancer. Surgery can be invasive and may require a long recovery time, and it may not be feasible for some patients depending on the site and extent of cancer [8]. Furthermore, these treatments can often be expensive and may not be affordable to all patients, particularly those in developing countries. As a result, there is a growing interest in developing alternative or complementary cancer treatments, such as immunotherapy, targeted therapy, and integrative medicine, that can address some of these limitations to improve patients’ lives [11].

## 2. Photodynamic Therapy

**Figure 1:**
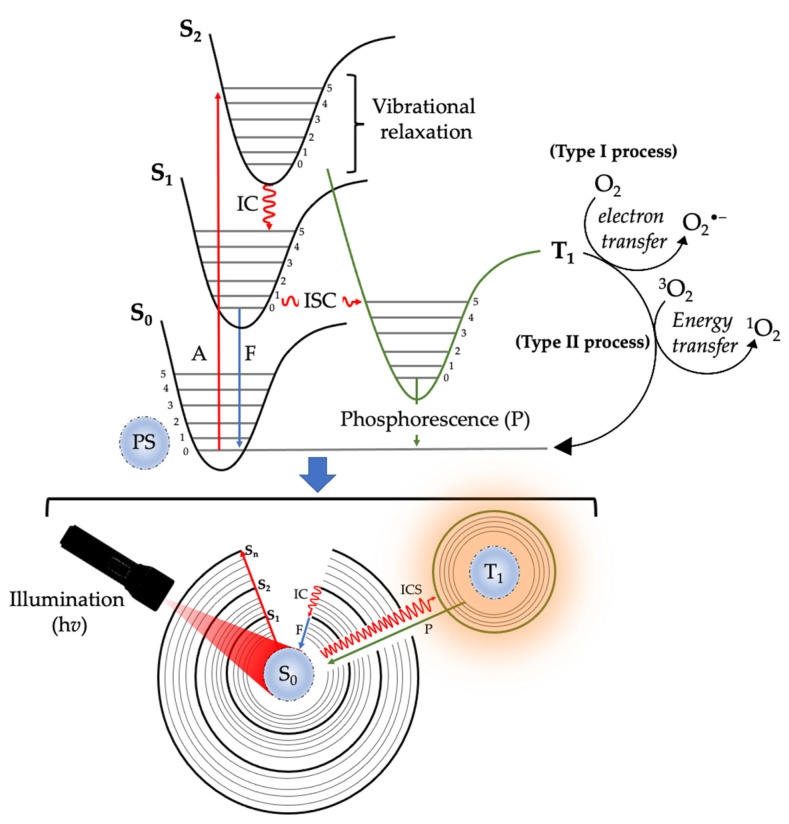
Schematic representation of the mechanism of action of photodynamic therapy. Excitation of light-illuminated photosensitizer from ground state to excited singlet states (S_1_ and S_2_), internal conversion (IC), intersystem crossing (ISC), and triplet excited state of a photosensitizer (T_1_) interacts with molecular oxygen (O_2_) in Type I and Type II pathways, thus leading to the formation of reactive oxygen species (ROS) and free radicals.

Photodynamic therapy (PDT) is a type of cancer treatment that uses a photosensitizer (PS) to create reactive oxygen species (ROS) by exposure to light, and these ROS can kill cancer cells. PDT has been applied to treat different kinds of cancer, including skin, lung, bladder, and oesophageal cancer [12]. One of the advantages of PDT is that it can selectively target cancer cells while sparing normal cells. This is because the PS is preferentially taken up by cancer cells and the light used to activate the PS can be precisely targeted to the tumour site [13]. This reduces the risk of damage to surrounding healthy cells, which is a common side-effect of conventional therapies such as chemotherapy and radiation therapy [14]. The ROS generated by PDT can also induce various biological responses, including immune system activation and inflammation which can contribute to the destruction of cancer cells [15]. As shown in Figure 1, the essential components for PDT include light, PS, and molecular oxygen. During PDT, the administered PS undergoes activation when it is exposed to a specific wavelength light, thus resulting in its transition from ground to an excited state [16]. Upon returning to the ground state, it releases energy that is transferred to oxygen, leading to the production of cytotoxic reactive oxygen species (ROS) such as free radicals and singlet oxygen [12]. Herein, we review the classification of PSs, PDT’s mechanism of action, highlight its limitations in cancer therapy, and the role of phytochemicals in PDT of cancer.

One of the primary limitations of PDT is the requirement for a light source that can effectively activate the PS. The depth of light penetration is limited, which restricts the use of PDT to surface tumours or tumours that can be reached by an endoscope or catheter [16]. Moreover, the PSs used in PDT can have some limitations, such as limited selectivity for cancer cells, potential for skin photosensitivity, and accumulation in non-targeted tissues, leading to off-target side effects [17]. In addition, PDT can induce the manifestation of other side effects e.g., pain, swelling, redness, and blistering at the site of treatment [18]. Overall, while PDT holds great promise as a cancer treatment modality, its clinical application is limited by several factors, including the depth of light penetration, the pH of the tumour microenvironment, and off-target side effects of the PSs.

### 2.1. Classification of Photosensitizers Used in PDT

The classification of therapeutic PSs involves a multifaceted process that assigns various PSs into specific classes. Multiple schemes may be used to classify PSs, depending on the context of the research and/or application. PSs can be classified based on their chemical structures, mechanism of action, and applications. Some of the common classes of PSs are the first, second, and third generation [16,19]. The first generation of PSs includes porphyrins and their derivatives, such as hematoporphyrin derivatives (HpD), which were among the first PSs used in PDT [20]. There is some evidence that HpD can be used for certain types of cancers, such as brain, cervical, endobronchial, oesophageal, bladder and gastric cancers. After purification, HpD was found to be ineffective for localizing tumours. However, modifications were made to the original structure by adding acetic-sulfuric acid mixtures to the HpD compound [21]. Clinical studies have revealed that photofrin has limitations, such as a complex composition and a low light absorption rate. The study conducted preclinical trials on mice models to assess the dosage of PDT, the level of apparent reacted singlet oxygen, and to predict the local control rate of tumours induced by radiation-induced fibrosarcoma [22].

The second generation of photosensitizers, such as benzoporphyrin derivatives (BPD) and mono-L-aspartyl chlorin e6 (NPe6), are synthetic compounds that offer significant improvements over the first generation. These improvements include a better composition and structure, increased photosensitivity, a wider absorption spectrum, and improved tissue selectivity. The first-generation photosensitizers had complex components, which led to poor tissue selectivity and slow response times in photodynamic damage intensity. Examples of second generation photosensitizers include benzoporphyrin, purpurin, texaphyrin, phthalocyanine, naphthalocyanine, and protoporphyrin IX [23]. Other photosensitizers, such as chlorins and phthalocyanines, have also been developed for PDT applications [24]. In 1984, J.S. McCaughan et al. employed PDT as a therapeutic approach for treating oesophageal cancer. Similarly, Balchum et al. utilized PDT to manage lung cancer patients in the same year. One year later, Hayata et al. applied PDT as a treatment option for patients with gastric carcinoma. These studies demonstrate the use of PDT in cancer therapy during the mid-1980s [16,25,26]. As all these studies indicated favourable outcomes in patients with early-stage cancers, PDT was suggested as a viable alternative for patients who could not undergo surgery due to other medical complications [16]. During the mid-1990s, J.C. Kennedy and his colleagues published their findings on the effective treatment of skin disorders using topically applied 5-ALA. Since then, the use of PDT that involves the localized administration of 5-ALA has become increasingly common in the detection and treatment of superficial lesions [27].

The third generation of photosensitizers includes nanomaterial-based photosensitizers, such as gold nanoparticles which have shown promise in improving the efficacy and specificity of PDT. As shown in Table 1, gold nano-clustered hyaluronan nano-assemblies were used to target the orthotopic breast tumour model [28]. To enhance absorption of chlorin E6 (Ce6) by the tumour as well as to increase ROS production, Ce6 was incorporated into nanoparticles via ion complexation. In addition to improving cancer imaging and treatment, Ce6 was also developed to encapsulate gold vesicles due to its strong NIR absorption (in the near-infrared range between 650 and 980 nm) [29,30,31]. Clinical trials are currently underway to investigate more selective and potent sensitizers. The effectiveness of PDT could be enhanced by various factors such as the use of new drugs, improved localization techniques, and the implementation of better protocols and equipment [32,33]. 

A fascinating approach involves modifying a photosensitizer by attaching it to a cytotoxic warhead, which offers both targeted tissue distribution and additional chemotherapeutic effects. This dual functionality proves especially advantageous as photodynamic therapy (PDT) typically treats a limited area in a single application and is less effective against metastatic cancer [34,35]. By incorporating a cytotoxic warhead, the conjugate can also target and eliminate cancer cells in non-illuminated regions. Consequently, this conjugate acts synergistically as a bimodal agent, possessing both photodynamic and chemotherapeutic properties. Research has demonstrated that combining a photosensitizer with a chemotherapeutic drug in the same treatment regimen exhibits significantly greater potential compared to using a single approach [36]. Transition metal complexes are widely recognized for their remarkable effectiveness against tumours. This has led to the development of numerous potent compounds, some of which have become integral components of contemporary chemotherapy treatments [37,38]. Typically, the antitumor metal complexes are combined with a photosensitizer (PS) to form conjugates that exhibit amphiphilic properties and excellent solubility in water. This is primarily attributed to the inclusion of a hydrophilic metal-containing component [38].

**Table 1:** General classification and examples of photosensitizers used in cancer therapy.

PS Name	Class	Chemical Structure	Application	Wavelength	Ref.
Hematoporphyrin (HpD)	Photofrin	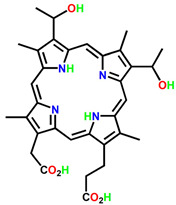	Brain, cervical, endobronchial, oesophageal, bladder and gastric cancers	30 nm	[21]
Benzoporphyrin derivative monoacid (BPD-MA)	Verteporfin	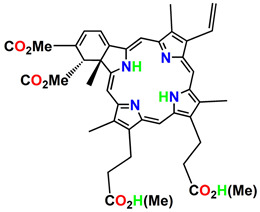	Basal cell carcinoma	689 nm	[23]
Temoporfin (m-THPC)	Foscan	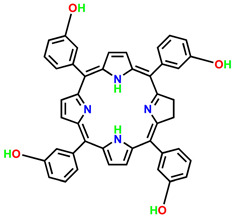	Head and neck, prostate, and pancreatic cancers	652 nm	[39]
5-aminolevulinic acid (5-ALA)	Levulan	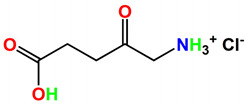	Basal cell carcinoma, brain, head and neck and bladder and gynaecological cancers	375–635 nm	[27]
3-[1′-hexyloxyethyl]-2-devinylpyropheophorbide-a (HPPH)	Photochlor	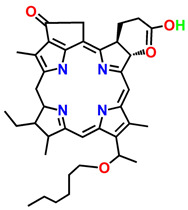	Basal cell carcinoma	665 nm	[40]
Gold Nano clustered Hyaluronan Nano-Assemblies	Nano assemblies	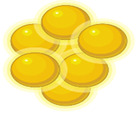	Orthotopic breast tumour model	-	[28]
Chlorin E6 (Ce6) + Upconversion nanoparticles	Nanoparticles	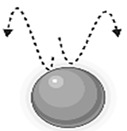	THP-1 macrophages	980–405 nm	[29]
ICG-loaded nanospheres coated with chitosan	Nanoparticles	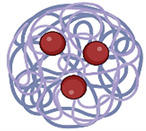	Coronavirus disease 2019, Ebola, and AIDS	800–805 nm	[41]

### 2.2. Application of Phytochemicals in PDT

**Figure 2:**
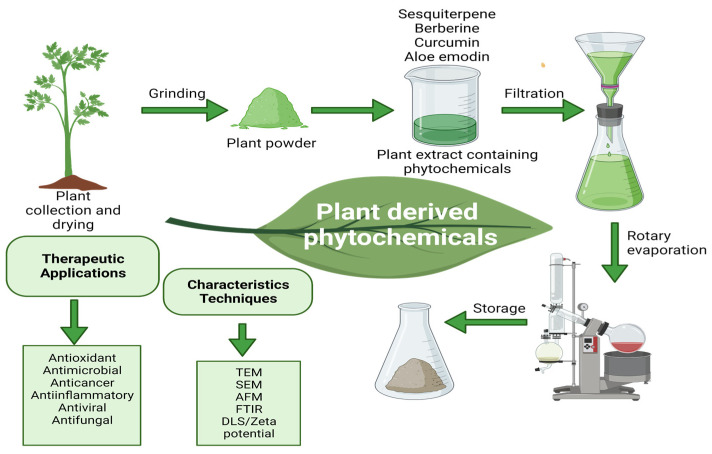
The extraction and characterization techniques for phytochemicals. Detailed methods involved in the extraction, isolation and characterization of phytochemicals and bioactive compounds from plants along with various pharmacological activities are listed.

Phytotherapy, or herbal medicine, is the use of plant extracts or phytochemicals for medicinal purposes or to manage health conditions [42,43]. The use of plants for medicinal purposes dates back thousands of years, and it continues to be an important aspect of healthcare in many cultures around world [44]. Phytotherapy has been gaining attention in combination with PDT due to its various benefits, including lower toxicity, higher selectivity, and a broad range of phytochemicals with diverse mechanisms of action [45,46]. The use of natural products, including plant extracts and phytochemicals, has been shown to enhance the therapeutic efficacy of PDT by increasing the production of reactive oxygen species (ROS) and inducing apoptosis in cancer cells [47,48]. Moreover, some plant-derived compounds, such as curcumin and resveratrol, have been found to possess photoprotective effects, preventing damage to healthy cells during PDT [49]. Several studies have investigated the potential of plant extracts in enhancing the efficacy of PDT. For instance, the combination of curcumin and PDT has been shown to induce cell death in pancreatic cancer cells and enhance the production of ROS [50]. Figure 2 shows the phytochemical extraction and characterization techniques.

Phytocompounds, which are naturally occurring compounds found in plants, offer sever advantages over conventional therapies. Firstly, they are generally considered to be safer and have fewer adverse effects compared to synthetic drugs. Many phytocompounds have been used for centuries in traditional medicine with minimal side effects [51]. Secondly, phytocompounds have a broad range of therapeutic effects due to their complex chemical structures, which can interact with various cellular targets. For example, flavonoids have been shown to have anti-inflammatory, anticancer, and antiviral activities [52]. Thirdly, phytocompounds are relatively inexpensive to produce and are easily accessible, particularly in developing countries where traditional medicine is still widely used. Lastly, phytocompounds have the potential to act synergistically with other phytocompounds or with conventional therapies to produce better therapeutic outcomes. For example, the combination of curcumin and chemotherapy has been shown to be more effective in treating cancer than chemotherapy alone [42,53]. 

*D. anomala* is a perennial herb distributed throughout Sub-Saharan Africa. The aerial parts of this plant have been the subject of analysis for the detection of various phytochemical compounds. The roots and leaves of *Dicoma anomala* (*D. anomala*) have been utilized in Africa for treating a variety of illnesses, and its extracts are known to possess anticancer properties that are particularly useful for treating breast and lung cancers. Some of the compounds found in *Dicoma* species sesquiterpenes listed in Table 2 have been identified as having anticancer effects [54,55]. In 2006, Steenkamp and Gouws conducted a study to evaluate the cytotoxic effects of plant extracts commonly used for cancer treatment in South Africa. Their findings indicated that aqueous extract obtained from the *D. capensis* plant exhibited anticancer properties against three different types of breast cancer cell lines, including MCF-7, MDA-MB-231, and MCF-12A [56]. In a study conducted by Tripathy et al., the researchers examined the potential antiproliferative effects of silver nanoparticles synthesized from the roots of *D. anomala* Sond. They evaluated these effects *in vitro*, specifically against MCF-7 breast cancer cells and NF54 parasitic strains [57]. Additionally, the study also investigated aqueous root extracts from *D. anomala* to explore their potential in reducing postprandial hyperglycaemia and modulating the activities of carbohydrate-metabolizing enzymes [58,59]. The pharmacological properties of *D. anomala* have been investigated for their potential in the treatment of various diseases. Extracts from *D. anomala* have been shown to possess a range of beneficial properties, including antibacterial, anti-inflammatory, antiviral, antioxidant, anticancer, and anti-plasmodial activities [58]. The anticancer properties of *D. anomala* have attracted the attention of researchers in the field of biomedicine, leading to a focus on its potential therapeutic applications. *Aloe vera (A. vera)*, a gel-bearing plant from the Xanthorrhoeaceae family, is widely distributed in various continents, although it is native to Africa. The gel found in the leaves of this plant is known to contain a wide range of beneficial compounds such as vitamins, minerals, amino acids, enzymes, mono- and polysaccharides, anthraquinones, phenolics, saponins, lignin, and salicylic acid. These phytoconstituents are responsible for various biological properties. The major secondary metabolites in *A. vera* are anthraquinones and tricyclic aromatic quinines [60,61,62]. Emodin and aloe-emodin have been found in *A. vera* to possess PDT effects through Type-I and Type-II reactions [63]. *Berberis aristata* is a plant with medicinal properties that is native to India and Nepal. It is predominantly found in the Himalayas and Sri Lanka, and is commonly used in Ayurvedic medicine to treat a range of health issues such as diarrhoea, jaundice, skin disorders, syphilis, chronic rheumatism, and urinary bladder disorders [64,65].

In Indian traditional medicine, *Curcuma longa* from the Zingiberaceae family has been historically utilized to treat a variety of ailments including infections and inflammation, as well as hepatic, gastric, and blood disorders. Curcumin is a phytochemical of *curcuma longa* used for the treatment of prostate, colorectal, breast, pancreatic, head and neck cancers as shown in Table 2. Another plant, *Ficus religiosa*, belonging to the *moraceae* family, is originally from the sub-Himalayan region, Bengal, and central India, but its cultivation has led to its widespread distribution worldwide. This plant has been traditionally utilized in herbal medicine to treat various health conditions related to the central nervous and endocrine system, gastrointestinal tract, and the reproductive and respiratory systems [66]. The phytochemical furanocoumarin is used for ovarian and breast cancer treatment. *Ipomoea mauritiana*, a plant commonly used in Ayurvedic and Folkloric medicine, has shown potential medicinal properties. The tuberous root of *I. mauritiana* displays multiple desirable characteristics. It has a sweet flavour and provides a refreshing sensation. This root is known for its appetizing effects, ability to promote lactation, and rejuvenating properties. Moreover, it acts as a stimulant, aids in digestion, and serves as a general tonic. Its composition consists of various phytochemicals, such as taraxerol, taraxerol acetate, β-sitosterol, scopoletin, 7-O-β-D glycopyranosyl scopoletin caffeoyl glucose, and 5-methoxy-6,7-furanocoumarin. Studies suggest that PDT utilizing furanocoumarins may be an effective treatment for autoimmune disorders and skin diseases [67,68]. Scopoletin, one of the phytochemicals of *I. mauritiana*, is used for the treatment of liver, lungs, skin, and breast cancer. *Rubia cordifolia*, a significant plant in Ayurveda, is utilized as a blood purifier and diuretic with vasodilating effects, as well as having diverse pharmacological benefits, including antiplatelet, antioxidant, calcium-channel-blocking, antidiabetic, and antistress properties. Its traditional use also includes treating ulcers, urinary discharges, jaundice, leukoderma, and piles [69]. As shown in Table 2, the phytochemical Rubiadin from *Rubia cordifolia* is used for the treatment of cervical and larynx cancers. In conclusion, the advantages of phytocompounds over conventional therapies and photosensitizers include their safety, broad range of therapeutic effects, affordability, and potential synergistic effects with other therapies.

**Table 2:** Summary of plant derived phytochemicals used in cancer studies.

Medicinal Plants	Phytochemicals	Application	Ref.
*Dicoma anomala*	Sesquiterpenes	Breast, lung, prostate cancers	[70]
*Aloe vera*	Aloe emodin, Emodin	Head and neck cancer, glioblastoma, colon, breast cancer, and gastric carcinoma	[63,71]
*Curcuma longa*	Curcumin	Prostate, colorectal, breast, pancreatic, and head and neck cancers	[72,73]
*Ipomoea mauritiana*	Scopoletin	Lung, liver, skin and breast cancer	[74,75,76]
*Berberis aristate*	Berberine	Breast and colorectal cancer	[77,78]
*Ficus religiosa*	Furanocoumarin	Breast and ovarian cancer	[79,80]
*Rubia cordifolia*	Rubiadin	Human cervical, and larynx cancer	[69,81,82,83]

Green photodynamic therapy (PDT) is an emerging field that harnesses the power of plant-derived phytochemicals and nanoparticles for therapeutic purposes. While it offers great potential, several challenges and limitations must be addressed for its successful implementation [84]. Nanoparticles used in green PDT can interact with biological systems, potentially leading to unintended side effects. It is essential to comprehensively understand the interactions between nanoparticles and cells/tissues to ensure their safety. Studies have focused on evaluating nanoparticle toxicity, biodistribution, and long-term effects [85]. The integration of nanotechnology in medicine raises regulatory and ethical concerns. Stringent regulations are necessary to ensure the safety and efficacy of green PDT-based therapies. Ethical considerations involve addressing issues such as informed consent, privacy, and equitable access to nanomedicine [86].

#### 2.2.1. Nanotechnology

Nanotechnology is a scientific and engineering discipline dedicated to the design and manipulation of materials and devices at the nanometre scale, aiming to achieve functional organization at this tiny level. This means that the size of the smallest unit in at least one dimension is one billionth of a meter. When dealing with materials or devices at incredibly small scales, it is essential to consider the behaviour of individual molecules and groups of molecules to comprehend their properties. By controlling the molecular structure of these materials, scientists can manipulate their macroscopic chemical and physical properties [87]. The term ‘nanotechnology’ was first introduced by physicist Richard Feynman in his 1959 talk “There’s Plenty of Room at the Bottom” [88]. Since then, the field has grown exponentially, with significant contributions from researchers all over the world. Scientists consider silicon to be a highly promising nanomaterial for biomedical applications, particularly in the areas of bioimaging and disease treatment, compared to other nanomaterials [89]. Among silicon-based materials, Mesoporous silica nanoparticles (MSNPs), exhibit exceptional physical and chemical properties that make them highly attractive for biomedical applications. As a result, they are considered a promising new class of inorganic materials in this field [90]. In the 1960s, a British scientist named Bangham discovered lipid nanoparticles, which are made up of one or more lipid bilayers [91]. The hydrophilic part of the phospholipid molecule faces the surrounding water, while drugs that are soluble in fat can be incorporated into the inner, hydrophobic part of the lipid nanoparticles [83]. Liposomes, depicted in Table 3, are commonly employed for the delivery of chemical drugs, genes, and siRNAs [83,92]. However, their mechanical stability is limited due to their thin membrane, resulting in a higher likelihood of drug leakage [93]. To enhance the effectiveness of liposomes in drug delivery, researchers have devised various techniques to address their limitations. These methods involve modifying the composition of phospholipids and introducing new components to enhance stability and reduce drug leakage. For instance, incorporating cholesterol can increase the membrane stiffness of liposomes, thereby improving their performance [94]. Micellar nanoparticles are created through the self-assembly of surfactants or amphiphilic block copolymers when their concentration surpasses a critical threshold in an aqueous solution. These particles are typically no larger than 200 nm in size [95].

The broad range of applications for polymer-based nanoparticles in biological preparations can be attributed to their versatility in synthesis. These nanomaterials possess a notable responsiveness to various stimuli encountered within the body, including enzymes and pH levels [96]. Most nanoparticles used for siRNA are cationic nanoparticles since siRNA is negatively charged, compressing it into delivery systems that can contribute to its uptake by cells. While cationic nanoparticles have high siRNA loading efficiency, their high charge may cause more toxicity to normal cells. Materials that are noncationic are likely to be a better choice in this situation [91,97]. The nanosphere is also used as a vehicle for drug delivery, as shown in Table 3. Spherical nanoparticles that are concentrically structured consist of a gold nanosphere coated with silica and surrounded by an outer layer of gold. These nanoparticles exhibit optical resonances that can be precisely adjusted based on their geometric properties, all while maintaining a compact size below 100 nm [98]. Gold nanorods are nanoparticles with an elongated shape, and their extinction spectra display two distinct plasmon resonances, as discussed in Table 3. The compact size of gold nanorods can be advantageous for photothermal therapies due to their ability to easily penetrate tissues and the permeable blood vessels found in tumours [99].

Gold nanoparticles (AuNPs) are a type of material that have at least one dimension with a size of less than 100 nm, and are used for drug delivery as shown in Table 3. Solid lipid nanoparticles (SLNs) are another type of lipid-based nanoparticle extensively utilized for drug delivery purposes [100]. They serve a significant role in the field. SLNs are sub-micron carriers designed to transport drugs, comprising of stable and biodegradable lipids with a high melting point, ensuring they remain solid at room temperature. These spherical particles typically range in size from 50 to 1000 nm [101,102,103]. Upconversion nanoparticles (UCNPs) are renowned for their remarkable optical characteristics, playing a significant role in advancing the field of biophotonics when combined with optical bioimaging technology [104]. In contrast, polyamide-amine (PAMAM) exhibits fundamental dendrimer traits, including well-defined molecular structure, hydrophobic cavities within the molecule, and the ability to regulate molecular size [105]. These characteristics make PAMAM an effective encapsulating agent for nucleic acids and other therapeutic drugs, thus constituting a highly suitable carrier for targeted therapy and diagnostic drugs [106,107]. In recent advancements, drug delivery units incorporating silver nanoparticles (AgNPs) have been developed to improve the effectiveness of treatments and have shown promising results in live subjects. These units utilize hybrid molecular structures that contain AgNPs and are designed to respond to optical, thermal, and pH changes, allowing targeted delivery for various conditions such as cancer, inflammation, and infections. AgNPs have gained popularity as drug carriers due to their excellent biocompatibility and the ability to modify their surfaces and optical properties through easily accessible synthesis methods [108].

**Table 3:** Examples of different nanoparticles used in drug delivery system and therapeutics.

Nanoparticle Name	Structure	Size	Excitation Wavelength (nm)	Application	Ref.
Mesoporous silica nanoparticles (MSNs)	** 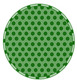 **	30–300 nm	804–815 nm	Drug delivery, bioceramics, pharmaceutics, and biomedicine	[109,110,111]
Liposomes	** 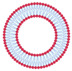 **	0.025–2.5 μm	-	Pharmaceutics, and drug delivery	[112]
Micelle	** 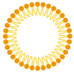 **	5–200 nm	420 nm	Cancer treatment, skin treatment, eye treatment, head and neck cancer treatment, and drug delivery	[95,113]
Noncationic polymer nanoparticles	** 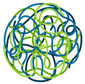 **	1–1000 nm	-	Drug delivery, theragnostic and bioimaging	[114]
Nanosphere	** 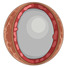 **	<100 nm	615 nm	Drug delivery	[100,115]
Gold nanoparticles (AuNPs)	** 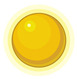 **	<100 nm	740 nm	Drug delivery	[100,116]
Nanorods	** 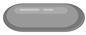 **	10–120 nm	700–800 nm	Electronic, optical, magnetic, and micromechanical devices	[117]
Solid lipid nanoparticles (SLNs)	** 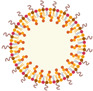 **	50–1000 nm	495–515 nm	Drug delivery	[103,118]
Upconversion nanoparticles (UCNPs)	** 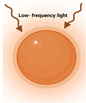 **	**-**	970 nm	Bio-photonics, and bioimaging	[104,119]
Polyamide-amine (PAMAM)	** 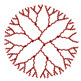 **	10–1000 nm	450 nm	Targeted therapy, diagnostic therapy, and drug delivery	[108,120,121]
Silver nanoparticles (AgNPs)	** 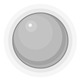 **	1–100 nm	239–314 nm	Drug delivery, cosmetics, health-care, anti-fungal, anti-bacterial and anti-inflammatory	[108,122,123]

#### 2.2.2. Therapeutic Applications of Nanotechnology

Nanotechnology has opened new possibilities in the field of medicine, especially in the development of therapeutics. One of the most promising applications of nanotechnology in medicine is targeted drug delivery. Nanoparticles can be engineered to selectively target diseased cells or tissues, which increases the efficiency and reduces the side effects of therapeutic agents [124]. Nanoparticles can also be designed to enhance the bioavailability of drugs and improve their pharmacokinetics. For instance, cancer cells are known to be highly vascularized, and they express unique biomarkers that can be exploited by nanoparticles to selectively target them [125]. Overall, the development of nanotechnology-based therapeutics is a rapidly evolving field that holds great promise for the treatment of various diseases. Nanoparticles can encapsulate drugs and selectively target cells or tissues, allowing for more effective and targeted delivery of therapeutics. For example, liposomes, solid lipid nanoparticles, and polymeric nanoparticles have been used to deliver chemotherapeutic agents to cancer cells, reducing systemic toxicity and increasing efficacy [125,126].

Nanoparticles can be engineered to enhance the contrast of medical imaging modalities such as magnetic resonance imaging (MRI), computed tomography (CT), and ultrasound. This can aid in the early diagnosis and monitoring of diseases such as cancer and cardiovascular disease [127,128]. Gene therapy has the potential to revolutionize the treatment of genetic disorders by enabling the direct correction of disease-causing mutations. Viral vectors are commonly used for gene delivery due to their high transfection efficiency. However, they can also elicit an immune response and have limited cargo capacity. Nanoparticles can be used to encapsulate viral vectors and protect them from immune recognition, as well as to increase their targeting specificity [129,130]. Non-viral vectors, such as liposomes and polymeric nanoparticles, have also been developed for gene delivery. These vectors are less immunogenic than viral vectors and can be designed to target specific cells or tissues. However, their transfection efficiency is often lower than that of viral vectors [131,132]. MSNs possess a large surface area and high pore volume, allowing for high drug loading and efficient encapsulation of photosensitizers [133,134]. MSNs can be easily functionalized with targeting ligands to improve specificity and selectivity, facilitating targeted delivery of the photosensitizer to cancer cells [135]. They exhibit excellent biocompatibility, low toxicity, and negligible immunogenicity, making them suitable for *in vivo* applications [136]. MSNs can suffer from limited stability under physiological conditions due to potential aggregation or degradation, which may affect their drug release kinetics [137]. The synthesis of MSNs can be complex and time-consuming, requiring precise control over particle size and morphology. In some cases, MSNs may induce an immune response or cause inflammation, necessitating careful consideration of their biocompatibility [134,138]. Micelles offer efficient encapsulation and solubilization of hydrophobic photosensitizers, enhancing their stability and bioavailability. The small size of micelles allows for improved tumour penetration and accumulation, leading to enhanced PDT efficacy [139,140]. Micelles often have limited drug loading capacity compared to MSNs, potentially leading to suboptimal therapeutic doses. The release of the photosensitizer from micelles may not be as controlled as in MSNs, which can impact the desired drug release kinetics [141]. Lipid-based nanoparticles, such as liposomes and lipid nanocarriers, offer excellent biocompatibility, controlled drug release, and the ability to encapsulate both hydrophilic and hydrophobic photosensitizers. They can be surface-modified for targeted delivery and improved cellular uptake [142,143]. Lipid-based nanoparticles may suffer from limited stability, premature drug release, and difficulties in large-scale production. Additionally, their relatively large size may hinder deep tissue penetration [124,144]. The development of the CRISPR/Cas9 system for genome editing has revolutionized the field of gene therapy. Nanoparticles have been used to deliver the CRISPR/Cas9 system to target cells, enabling the correction of disease-causing mutations [145,146]. RNA interference (RNAi) is a powerful tool for gene regulation that can be used to treat a variety of diseases. Nanoparticles can be designed to deliver siRNA to target cells, enabling the specific downregulation of target genes [147]. Gold nanoparticles possess excellent biocompatibility, ease of synthesis, and tuneable optical properties for enhanced light absorption and scattering. They can efficiently generate heat upon laser irradiation, leading to photothermal therapy in addition to PDT [148,149]. The disadvantage of gold nanoparticles is that they may induce potential cytotoxicity depending on their size, concentration, and surface modification. Additionally, their accumulation in certain organs may pose long-term health risks [150,151].

Although the field of gene therapy using nanotechnology is relatively new, it shows immense potential in addressing various genetic disorders. While additional research is required to comprehensively assess the safety and effectiveness of these methods, the advantages they offer are evident. By utilizing nanoparticles, it becomes possible to specifically target immune cells or tissues, facilitating the direct administration of therapeutic drugs to sites affected by inflammation or infection. For example, liposomes have been used to deliver anti-inflammatory drugs to inflamed tissues, reducing inflammation and promoting tissue repair [152]. They can also be used as biosensors to detect and monitor the immune response. For example, gold nanoparticles have been used to detect the presence of cytokines and other immune-signalling molecules in biological fluids [153]. Nanomaterials can also be used to promote tissue regeneration and repair. For example, scaffolds made of nanofibers can be used to support the growth and differentiation of stem cells, aiding in the repair of damaged tissues [154].

## 3. Green Nanotechnology

Green nanotechnology refers to the development of eco-friendly nanomaterials and nanotechnology-based processes. One example of green nanotechnology is the use of plant extracts as reducing agents to synthesize nanoparticles. The synthesis of nanoparticles using plant extracts has several advantages over traditional methods, including the use of non-toxic and renewable materials, and the ability to synthesize nanoparticles at room temperature and pressure [155]. In addition, green nanotechnology can also be applied to the development of sustainable and environmentally friendly energy sources. For example, nanocellulose-based materials have shown promise as sustainable and renewable materials for energy storage and conversion [156].

Green nanotechnology has garnered significant attention in recent years as a potential solution to address the environmental and health concerns associated with conventional nanotechnology. This approach offers a promising future perspective in developing safe and sustainable nanomaterials for various applications, including drug delivery, energy production, and water treatment. However, as with any emerging technology, the potential health risks associated with green nanotechnology also need to be evaluated. Nanoparticles, even those considered eco-friendly, can induce cell death through various mechanisms such as oxidative stress, apoptosis, and necrosis. Further studies are needed to understand the toxicity mechanisms of green nanomaterials and develop safe practices for their use [156].

Green nano-biotechnology refers to the process of creating nanoparticles or nanomaterials through biological means, utilizing microorganisms, plants, viruses, or their by-products such as proteins and lipids, with the aid of biotechnological tools. Nanoparticles produced through green technology exhibit numerous advantages over those manufactured using physical and chemical methods, considering various factors. For instance, green techniques eliminate the need for costly chemicals, consume less energy, and yield environmentally friendly products and by-products. The twelve principles of green chemistry have become a widely recognized guide for researchers, scientists, chemical technologists, and chemists worldwide, enabling the development of less hazardous chemical products [157]. Green nanobiotechnology presents a promising alternative pathway for producing stable nanoparticles that are biocompatible [158]. The typical procedure involves using dried plant biomass and metallic salts, where the plants act as bio-reducing agents and the salts serve as precursors. The antimicrobial and preservative properties of silver have been recognized for thousands of years. The biologically based synthesis of nanoparticles follows a bottom-up approach, relying on reducing and stabilizing agents. The process involves three main steps: selecting an appropriate solvent medium, utilizing an environmentally friendly and benign reducing agent, and employing a non-toxic material as a capping agent to stabilize the synthesized nanoparticles [159]. The researchers synthesized various chlorophyll derivatives and evaluated their photodynamic activities. They found that these derivatives exhibited excellent singlet oxygen generation and phototoxicity against cancer cells. In addition, many studies have also demonstrated the therapeutic potential of chlorophyll-based photosensitizing agents for tumour imaging and targeted therapy [160]. The researchers employed a plant-extract-mediated green synthesis method to produce various metallic nanoparticles including AuNPs with excellent stability and biocompatibility. The synthesized AuNPs were then used as carriers for a photosensitizer and showed enhanced photodynamic therapeutic efficacy both *in vitro* and *in vivo* [161].

### 3.1. Synthesis and Characterization 

Nanoparticles are tiny particles with dimensions typically ranging from 1 to 100 nm, and they have unique properties due to their small size and large surface area. The synthesis and characterization of nanoparticles are essential for their use in various fields such as medicine, electronics, and environmental remediation [162].

#### 3.1.1. Synthesis Methods 

Chemical reduction method is one common method for the synthesis of nanoparticles, which involves the use of reducing agents to convert metal ions into nanoparticles. This method has been extensively studied for the synthesis of metal nanoparticles, such as gold and silver nanoparticles [163]. Sol-gel is another method employed in the synthesis of nanoparticles; it involves the hydrolysis and condensation of metal alkoxides to form a gel. In this method, the synthesized gel is dried and calcined to produce the desired nanoparticles [164].

#### 3.1.2. Characterization Methods 

There are different characterization methods employed in nanotechnology. These methods aim to investigate the characteristics of nanoparticles. Examples of characterization methods for nanoparticles include transmission electron microscopy (TEM), scanning electron microscopy (SEM), X-ray diffraction (XRD), and Fourier-transform infrared spectroscopy (FTIR). These techniques provide valuable insights into the properties and structure of nanoparticles. These techniques can provide information on the size, shape, crystal structure, and chemical composition of nanoparticles [165].

##### Transmission Electron Microscopy (TEM)

Transmission electron microscopy (TEM) is a highly valuable method for analysing and characterizing nanoparticles. This technique utilizes a focused electron beam to examine thin samples, typically less than 200 nm thick, enabling the generation of high-resolution images that provide exceptional spatial resolution for nanoscale materials [166]. TEM allows for the examination of the crystalline structure of specific microscopic areas within crystalline materials with spatial confinement and focusing of the electron beam, which results in the detection of electron diffraction patterns. This technique enables researchers to analyse the crystallographic orientation of nanoparticles, defects, and grain boundaries in high resolution [167]. 

##### Scanning Electron Microscopy (SEM)

The scanning electron microscope works by detecting the secondary electrons that are emitted from the sample when it interacts with the electron beam. This allows for imaging of the surface of the sample [168]. SEM and TEM are both imaging techniques commonly used for the characterization of nanoparticles. In scanning electron microscopy (SEM), the use of lower beam energies is common for imaging samples. This choice of energy limits the depth to which the electron beam can penetrate, resulting in sensitivity only to the surface of the specimen. As a result, SEM is particularly valuable for examining the morphology of relatively thick samples (>100 nm) that cannot be adequately analysed using transmission electron microscopy (TEM). On the other hand, TEM provides higher-resolution imaging of the internal structure and composition of nanoparticles, but is limited to thin samples [165,169].

##### Atomic Force Microscopy (AFM)

Atomic force microscopy (AFM) is a type of scanning probe microscopy technique that enables probing and visualization of the surface, as well as several other force-related quantities, of objects with nanometre-sized or even atomic-sized dimensions [170]. AFM is a technique that uses a sharp tip attached to a cantilever to measure interactions with the surface of a sample. The cantilever may experience either a vertical or lateral deflection, or a change in amplitude, frequency, or phase of an oscillating cantilever, depending on the measurement mode used. There are three main modes of operation in AFM: contact mode, non-contact mode, and tapping mode. In contact mode, the tip remains in constant contact with the sample surface, and the deflection of the cantilever is caused by the repulsion of the tip and the surface atoms of the sample. This deflection can reveal information about the surface topography of the sample. Nanoparticle characterization is a diverse and intricate field that plays a crucial role in nanoscience. However, in the dynamic landscape of nanotechnology, the process of measuring and standardizing nanoparticles often lags behind the rapid advancements in new materials and their various applications. Despite the challenges, nanoparticle characterization is essential for understanding and optimizing the properties of nanoparticles for their various applications [171].

## 4. Conclusions

In conclusion, green nanotechnology offers an exciting prospect for sustainable and environmentally friendly nanomaterials. However, the potential health risks associated with green nanomaterials should be thoroughly investigated to ensure their safe and responsible use. Although synthetic drugs have been extensively studied for photodynamic therapy (PDT), there has been relatively little investigation into medicinal plant extracts or bioactive compounds obtained from plants, which are generally considered to be safer than synthetic chemicals. This approach using plant-based photoactive compounds for PDT is considered eco-friendly, and this review offers valuable insights for future research in this field. Ultimately, determining the scientific potential of using green photodynamic therapy with plant-based compounds may lead to new treatment alternatives for various chronic illnesses. Further, an advantage of PDT is that it can be carried out in low-resource settings and at a reasonable cost, which gives patients the chance to avoid more invasive surgical procedures. With carefully planned and executed clinical trials, it can be determined whether this potential benefit can be fully realized.

## Data Availability

Not applicable.
